# Retinal Microperimetry: A Useful Tool for Detecting Insulin Resistance-Related Cognitive Impairment in Morbid Obesity

**DOI:** 10.3390/jcm8122181

**Published:** 2019-12-11

**Authors:** Andreea Ciudin, Angel Michael Ortiz-Zuñiga, Enzamaria Fidilio, Diana Romero, Marta Sánchez, Marta Comas, Oscar Gonzalez, Ramon Vilallonga, Olga Simó-Servat, Cristina Hernández, Rafael Simó

**Affiliations:** 1 Institut de Recerca Vall d’Hebron, Universitat Autònoma de Barcelona (VHIR-UAB), Plaça Cívica, Barcelona 08193, Spain; angelmichaelortizzuniga@gmal.com (A.M.O.-Z.); efidilio@vhebron.net (E.F.); diana.romero@vhir.org (D.R.); olga.simo@vhir.org (O.S.-S.); cristina.hernandez@vhir.org (C.H.); 2 CIBER de Diabetes y Enfermedades Metabólicas Asociadas, Instituto de Salud Carlos III, Madrid 28020, Spain; 3 Department of Endocrinology, Vall d’Hebron University Hospital. Passeig Vall d’Hebron 119-139, Barcelona 08035, Spain; ma.san.pe@gmail.com (M.S.); m.comas@vhebron.net (M.C.); 4 Department of Surgery. Vall d’Hebron University Hospital. Passeig Vall d’Hebron 119-139, Barcelona 08035, Spain; osgonzal@vhebron.net (O.G.); rvilallonga@vhebron.net (R.V.)

**Keywords:** morbid obesity, cognitive impairment, retinal microperimetry, insulin resistance

## Abstract

Background: There is clear association between type 2 diabetes (T2D) and cognitive decline. Retinal microperimetry is a useful tool for detecting cognitive impairment in T2D. Morbid obesity (MO) has been associated with cognitive impairment. Insulin resistance (IR) seems a major determinant, but the data are unclear. The aim of this study was to evaluate the cognitive impairment in MO as well as the utility of retinal microperimetry in identifying these alterations. Methods: In total, 50 consecutive patients with MO were matched by age and gender with 30 healthy controls. All patients underwent cognitive evaluation (Montreal Cognitive Assessment Test-MoCA) and retinal microperimetry, using MAIA microperimeter 3rd generation. Retinal sensitivity and gaze fixation parameters were used for the evaluation of the analysis. Results: MO patients showed a significantly lower neurocognitive performance than the controls: MoCA score 24.94 ± 2.74 vs. 28.95 ± 1.05, *p* < 0.001. Cognitive function inversely correlated with the HOMA-IR (*r* = −0.402, *p* = 0.007). The AUROC for cognitive impairment using microperimetry was 0.807, CI 95% (0.592–0.947), *p* = 0.017. Conclusions: (1) Systemic insulin resistance is a major underlying mechanism accounting for the higher prevalence of cognitive impairment detected in young MO subjects. (2) Retinal microperimetry is a useful tool for identifying MO patients with cognitive impairment.

## 1. Introduction

Obesity represents a major public health problem and is associated with significant economic burden in the health systems of developed countries. This is mainly due to associated co-morbidities, type 2 diabetes (T2D) being the most important. At present, there is a clear relationship between type 2 diabetes and dementia [1,2]. Furthermore, T2D acts as an accelerator for cognitive decline [3] and shares with Alzheimer’s disease (AD) common pathophysiological pathways, such as inflammation, oxidative stress, and insulin resistance (IR) [4,5].

T2D has been associated with lower grey matter volumes and white matter disruption [6,7], as well as with impairment of perceptual speed, attention, and primary memory [8]. At present, there are no reported phenotypic indicators or reliable cost-effective screening tests for identifying T2D patients with cognitive impairment. Moreover, the diagnosis is based on neuropsychological tests whose complexity makes their incorporation into current standards of care for the T2D population unfeasible.

Recent evidence has shown that retinal neurodegeneration is an early event in the pathogenesis of diabetic retinopathy (DR) [9,10]. The retina is ontogenically a brain-derived tissue, and it has been suggested that it may provide an easily accessible and noninvasive way of examining the pathology of the brain [11,12]. Therefore, it seems reasonable to propose that the evaluation of retinal parameters related to neurodegeneration, such as retinal function, could be useful for identifying those patients with type 2 diabetes who have a high risk of developing AD. Several methods can be used to measure retinal function. Among them, fundus-driven microperimetry has emerged as a simple, noninvasive, and rapid test that can be used in clinical practice [13,14]. Microperimetry measures retinal sensitivity in terms of the minimum light intensity that patients can perceive when spots of light stimulate specific areas of the retina. Some recent studies have suggested that microperimetry is even more sensitive than multifocal electroretinography in detecting early functional changes of the retina [15]. Our group showed that retinal sensitivity assessed by fundus-driven microperimetry was related to brain neurodegeneration and could be a useful biomarker for identifying those patients at risk of developing AD [16]. Additionally, gaze fixation parameters evaluated during microperimetry improved the predictive value of retinal sensitivity in identifying cognitive impairment in subjects with T2D [17].

Obese patients with metabolic syndrome without hyperglycemia are more prone to showing global cognitive decline [18,19]. More recently, it has been demonstrated that cognitive decline even occurs at younger age, with the executive function and the perceptual speed related to visuospatial task being especially altered [20,21]. Several studies showed small brain volumes and white matter disruption in obese patients in comparison with normo-weighted counterparts [22,23,24]. Furthermore, similar brain structure findings related to AD (example cortical glyosis, hyppocampal atrophy, beta-amyloid deposits) were found in obese murine models [25,26]. Since similar alterations in brain structures are found in both T2D and obesity, it can be postulated that IR is a major underlying mechanism of the increased prevalence of AD reported in these populations. However, further clinical and basic research to support this concept is needed. 

The aim of the present study was to explore, by means of a pilot study, whether retinal sensitivity and gaze fixation assessed by microperimery could be useful for identifying obese patients with cognitive impairment and their relationship with IR.

## 2. Material and Methods

Between September 2018 and December 2018, a total of 50 consecutive patients who were attending the obesity outpatient clinic of Vall Hebron University Hospital for bariatric surgery (BS) evaluation were included in a case-control study as a part of the PREDIBAR trial- NCT 03784508. The inclusion criteria (cases) were: (a) Age between 18 and 60 years; (b) fulfill the criteria for BS; (c) previous accomplishment of the preoperative protocol for BS at our site; (d) ability to read and understand the specific questionnaires; and (e) written informed consent form. The exclusion criteria were: (a) Any form of diagnosed cognitive impairment; and (b) neurodegenerative diseases of the brain or retina (i.e., glaucoma) or cerebrovascular diseases. The control group comprised healthy persons with normal weight matched by age. Most of them were family members of patients included in the study from the same catchment area.

The study was approved by the local ethics committee and carried out in accordance with the Declaration of Helsinki.

All patients underwent complete physical, biochemical, and psychiatric evaluations as part of the preoperative protocol for bariatric surgery at our site. Cognitive function was evaluated using the Spanish version of Minimental State Evaluation (MMSE) [27] and the Montreal Cognitive Assessment Test (MoCA) [28]. The MoCA test evaluates several domains of cognitive function: Visual spatial executive (5 points), naming (3 points), attention (6 points), language (3 points), abstraction (2 points), delayed recall (5 points), and orientation (6 points). The required administration time is approximately 10 minutes. The maximum score is 30. The score was adjusted by age and education level (if the education level was <12 years, 1 point was added). We used a cut-off of 26 to define mild cognitive impairment as previously described [28].

Retinal sensitivity was evaluated by fundus-driven microperimetry MAIA 3rd generation after a previous pupillary dilation of a minimum of 4 mm. The standard MAIA test covers a 10-diameter area with 37 measurement points, and a red 1 radius circle was used as the fixation target. We employed an expert exam 4–2 strategy (Goldmann III size stimulus, background luminance of 4 asb, and maximum luminance of 1000 asb, with a 25 decibel (dB) dynamic range). For the evaluation of fixation, the MAIA microperimeter uses high-speed eye trackers (25 Hz) for the control of fixation losses and to register the fixation pattern. All fixation positions during the examination are used by the instrument to calculate the fixation indexes, P1 and P2, which represent the percentage of fixation points inside a circle of 2 and 4 degrees of diameter, respectively. Subjects were classified as having stable fixation if P1 was more than 75%, relatively unstable fixation if P1 was less than 75% and P2 more than 75%, and unstable fixation if both P1 and P2 were below 75%. Microperimetry also provides a more accurate estimation of the fixation pattern using the bivariate contour ellipse area (BCEA). This area is calculated as an ellipse that covers fixation eye positions and takes into account 1 or 2 times the standard deviation, including 63% (BCEA63) and 95% (BCEA95) of the fixation points. The area of this ellipse is calculated through major and minor axes, which are 2 orthogonal diameters, describing the extent of the fixation points (horizontal and vertical diameters) [29]. For retinal sensibility and fixation, data corresponding to the right eye was used, which was the first eye explored in all subjects.

### Statistical Analysis

To assess differences between groups, the Chi-square test for qualitative variables and ANOVA followed by DMS post-hoc tests for quantitative variables were used. To evaluate the correlations, Spearman’s correlation test and regression analysis were performed. Significance was accepted at the level of *p* < 0.05. The Bonferroni correction was used for multiple comparisons. Logistic regression was used to predict the ROC curves and the Chi-squared test for ROC area comparison. Statistical analyses were performed with SPSS 24.0 statistical package.

## 3. Results

The baseline demographical and laboratory data as well as the neurocognitive and psychological evaluation of obese patients and healthy controls included in the study are shown in Table 1. It should be noted that as per the protocol requirements, we did not find any difference in age.

We did not find differences in the results of MMSE between obese and control subjects. By contrast, a significant difference in the global cognitive function evaluated by MoCA was observed (Table 1). The main cognitive domains affected in the obese group were the executive function, attention, and recent memory (delayed recall) (Table 2).

Regarding microperimetry assessment, we found no significant differences between the obese patients and controls in terms of retinal sensitivity. By contrast, we found significant differences in all the parameters related to gaze fixation: P1, P2, BCEA63, and BCEA95 (Table 3).

**Figure 1:**
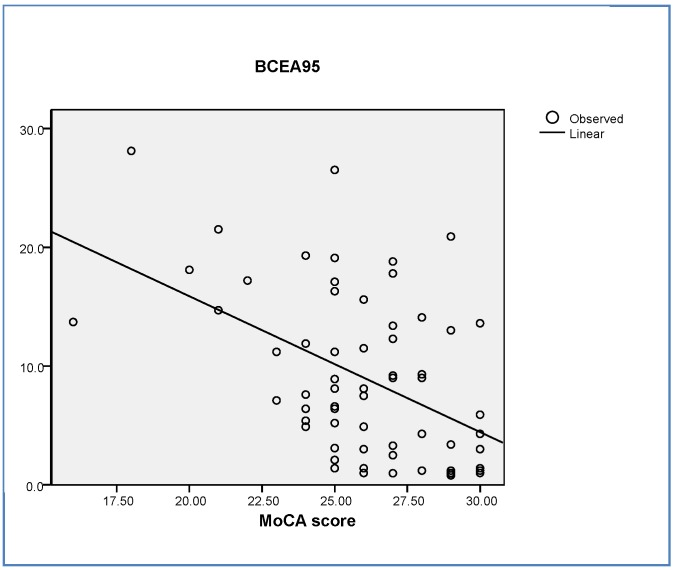
Correlation between BCEA95 and MoCA. BCEA95: 95% bivariate contour ellipse area. MoCA: Montreal Cognitive Assessment test.

The BCEA95 parameter, which is the most integrative parameter of gaze fixation, was significantly correlated to the MoCA score (R = 0.512, β = −0.510, *p* < 0.001) (Figure 1).

**Figure 2:**
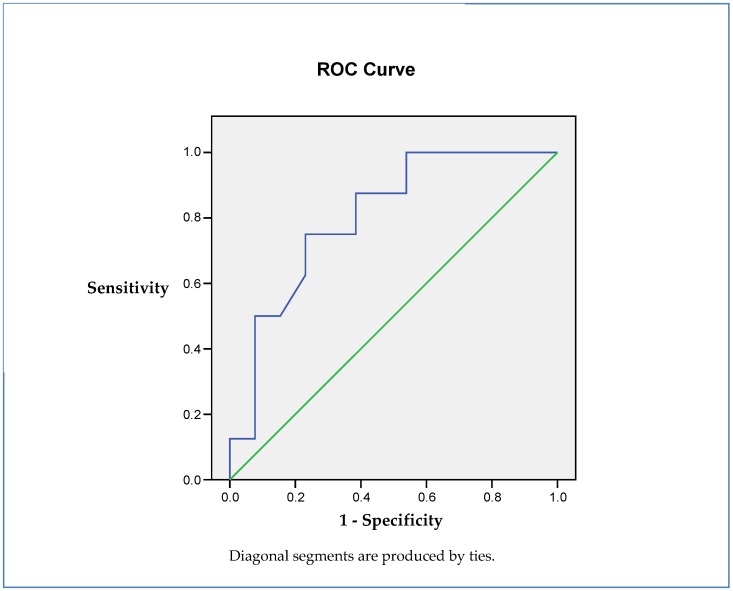
AUROC BECA95 and cognition (MoCA). BCEA95: 95% bivariate contour ellipse area. MoCA: Montreal Cognitive Assessment test.

Additionally, the BECA95 parameter was able to predict the cognitive status of the patients evaluated in the study by means of the MoCA test, showing an AUROC of 0.807 (CI 95% (0.592–0.947), *p* = 0.017) (Figure 2).

### Insulin Resistance (IR) as an Independent Predictor of Cognitive Status

The characteristics of obese subjects regarding the presence of diabetes are displayed in Table 4. We did not find significant differences in the demographic characteristics, cognitive function, retinal sensitivity, or gaze fixation between groups. As expected, HOMA-IR and HbA1C levels were significantly higher in obese subjects with diabetes.

Regarding antidiabetic treatment, 35% were on a diet only, 52% of the patients received one drug (metformin), and 13% received two drugs (metformin plus GLP-1 receptor agonists). No patient was on insulin treatment. 

Since obese patients presented poor cognitive scores using MoCA, we wanted to explore the variables accounting for this finding. For this purpose, a multiple regression analysis, including as variables age, gender, T2D status, HbA1c levels, BMI, and HOMA-IR, was performed. We found that HOMA-IR was the only independent predictor of cognitive impairment as reflected by Table 5.

**Figure 3:**
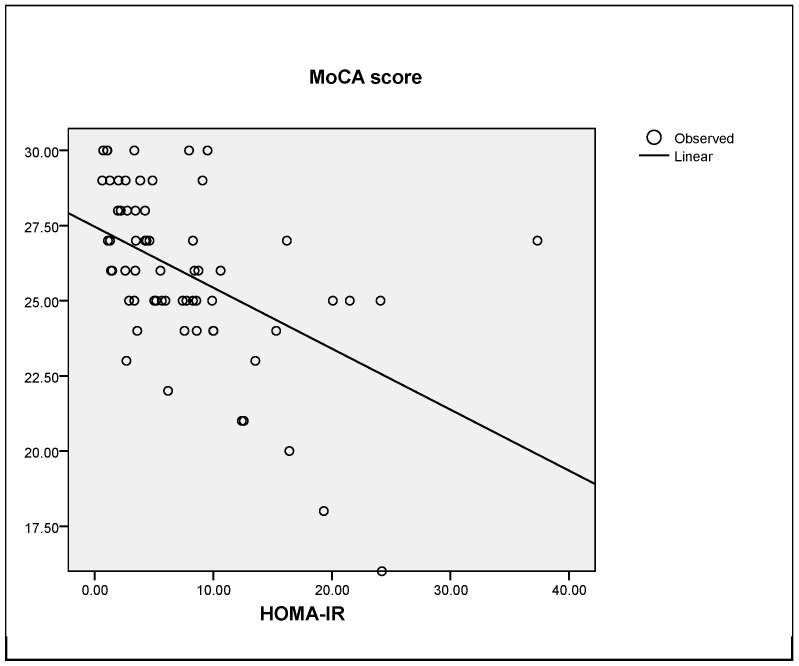
Correlation between MoCA score and HOMA-IR. MoCA: Montreal Congnitive Assessment test. HOMA-IR: Homeostatic model assessment for insulin-resistance.

In fact, the cognitive function evaluated by the MoCA score was correlated with the insulin resistance state evaluated by HOMA-IR (R = 0.402, *p* < 0.0001) as reflected in Figure 3.

**Figure 4:**
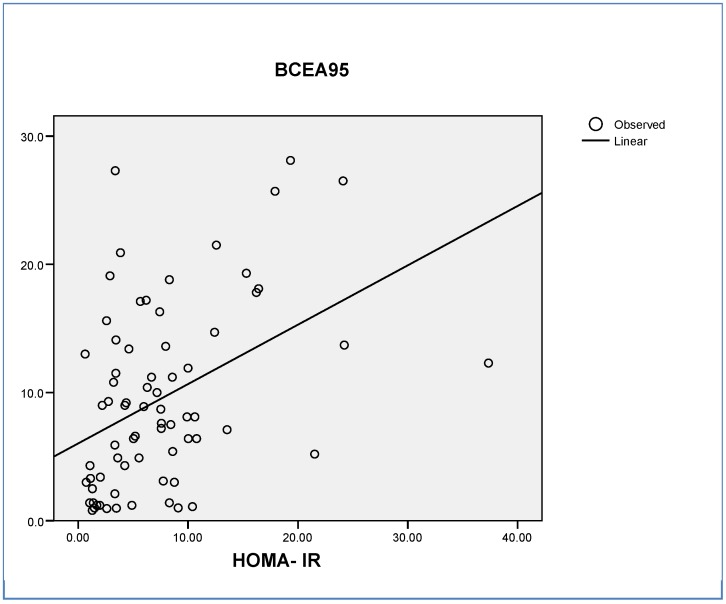
Correlation between BCEA95 and HOMA-IR. BCEA95: 95% bivariate contour ellipse area. HOMA-IR: Homeostatic model assessment for insulin-resistance.

Finally, we found a significant correlation between BCEA95 and HOMA-IR (R = 0.411, β = 0.470; *p* < 0.001) (Figure 4).

## 4. Discussion

In this study, we found a significant reduction in the cognitive function in young morbidly obese patients, when compared to their counterparts with normal weight, regardless of the presence of diabetes. In addition, we found that cognitive impairment assessed by the MoCA score adjusted by age correlated with the insulin resistance state evaluated by HOMA-IR. Furthermore, the assessment of gaze fixation by microperimetry was significantly correlated to the MoCA score and the HOMA-IR. Overall, our results suggest that IR could be a primary underlying mechanism accounting for both cognitive impairment related to obesity and gaze fixation abnormalities.

For the cognitive function evaluation, we used the MMSE for screening and the MoCA test. The latter can be very useful for early detection of mild cognitive impairment and dementia [28] and we found a significantly lower score in obese patients in comparison with controls. It should be noted that the MMSE score was within the normal range in all cases and no differences were found in our study in the MMSE score between cases and controls. This finding suggests that MMSE is not useful for detecting early cognitive impairment in young obese patients. Regarding the cognitive domains explored by MoCA, we found significant reductions in the obese patient group in the visual spatial executive function, attention, and delayed recall. These findings agree with recent data obtained in obese children and younger adults [20,21]. Nevertheless, in these studies, IR was not considered in the analyses of the results.

There are some previous studies suggesting systemic IR as a major determinant in the cognitive impairment in obesity. For instance, the Rotterdam study [29] showed that either the doubling of basal insulin levels or the presence of IR was associated with a 40% increased likelihood of converting to AD at a 3-year follow-up. Additionally, Rasgon et al. [30] showed a negative correlation between IR and hippocampal volume cognitive performance in middle-age women. Other authors found that higher fasting insulinemia was associated with less hippocampal and prefrontal gray matter in obese adolescents and young adults [31].

There is a clear relationship between T2D and cognitive impairment [3,4]. Clinical studies showed that T2D is associated with smaller brain volumes in grey matter [32]. These structural network abnormalities were related to the slowing of the information-processing speed [33]. Interestingly, brain damage and white matter disruption have been reported in the IR stage without overt diabetes [34]. These structural abnormalities could be involved in early cognitive impairment, in particular in perceptual speed, attention, and primary memory [34]. These studies were performed in middle-aged or older adults whereas we found similar results in terms of cognitive impairment in younger obese subjects. It should be noted that we did not observe differences in the cognitive status between obese subjects with and without diabetes, but IR was an independent predictor of cognitive decline. Taken together, these findings strongly suggest that the pathways related to insulin signaling rather than blood glucose levels play a key role in the development of cognitive impairment in the natural history of diabetes.

The impairment of insulin signaling (leading to insulin resistance) in the brain plays an essential role in the pathogenesis of dementia and precedes cognitive dysfunction and pathological alterations even by decades. Insulin signaling is essential for neuronal survival, as well as for learning and memorizing. In fact, in animal models, impaired insulin signaling precedes Aβ accumulation [35,36]. Once formed, Aβ oligomers deplete insulin receptors from the surface membrane of neurons, thereby contributing to insulin resistance [37], and produce an aberrant phosphorylation of insulin receptor substrate (IRS). This reduces the normal pro-survival signaling in neurons and promotes their death by apoptosis [38]. It should be noted that most of these studies focus on brain insulin resistance, rather than systemic IR. By contrast, in the present study, HOMA-IR (a measurement of systemic IR) was independently associated with the MoCA score. This raises the question of whether insulin-sensitizer drugs could be useful in preventing or arresting cognitive decline. In this regard, Luchsinger et al. [39] showed a slight arrest in cognitive decline related to lowered fasting insulin levels in middle-aged (65 years) non-diabetic patients with obesity (BMI 31 kg/m^2^) after 12 months of treatment with metformin. Additionally, Alosco et al. [40] showed a significant improvement in the cognitive function in young MO at 12 months follow-up after BS.

Another interesting finding in our study was that the gaze fixation parameters (ex. BECA 95) were correlated with both the HOMA-IR and cognitive function. In fact, the AUROC of BCEA95 for identifying young morbidly patients with cognitive impairment was 0.807 (CI 95%: 0.592–0.947). We previously reported that retinal sensitivity evaluated by retinal microperimetry was able to identify T2D subjects with cognitive impairment [16]. It should be noted that in this previous study, the T2D patients with Alzheimer’s disease and with mild cognitive Impairment were not obese but were insulin resistant. The patients presented a BMI of 27.72 ± 3.79 kg/m^2^ and 28.82 ± 4.48 kg/m^2^, respectively. Furthermore, the predictive value in identifying those T2D subjects with mild cognitive impairment was significantly increased by adding fixation parameters to the retinal sensibility [17]. This finding could be attributed to the fact that the brain areas involved in retinal fixation are not the same as in retinal sensitivity. Therefore, the combined test (retinal sensitivity + gaze fixation) results in an improvement of the efficiency of microperimetry in detecting MCI due to the examination of different neural circuits. In this regard, retinal sensibility relies on the retina and the neural pathway of vision. The first station of the optic tract is the lateral geniculate body of the thalamus, which is a major visual processing region in the brain and plays a crucial role in relaying information from the retinal ganglion cells to the primary visual cortex [41]. By contrast, the superior colliculus and the parietal and frontal cortex play an essential role in gaze fixation. There is evidence that both the geniculate body and the superior colliculus are affected by AD [42,43,44].

We have found that gaze fixation is impaired earlier than retinal sensitivity in obese patients. There are several reasons that could explain this finding, but perhaps the fact that gaze fixation depends on white matter complex connectivity networks, which are impaired earlier in subjects with IR, could be one of the most important [45,46].

To the best of our knowledge, this is the first study that provides a clinical link between systemic insulin resistance in obese subjects, cognitive impairment, and abnormal gaze fixation assessed by microperimetry. Our results suggest that retinal microperimetry could be a useful tool in identifying early cognitive impairment in MO patients. Further studies are needed to validate our results and also to evaluate the possible role of retinal microperimetry in monitoring the evolution of cognitive impairment and its relationship with IR after BS or pharmacological interventions.

## Figures and Tables

**Table 1:** The baseline characteristics of the subjects included in the study.

Characteristics	Obese Patients	Controls	*p*
*N*	50	30	NA
Gender (females %)	71.12	70.45	n.s.
Age (years)	45.88 ± 10.23	41.17 ± 12.33	n.s.
BMI (kg/m^2^)	44.54 ± 5.53	22.99 ± 3.51	<0.001
HOMA-IR	6.43 ± 2.64	1.34 ± 0.68	<0.001
HbA1c (%Hb DCCT)	5.96 ± 1.15	5.24 ± 0.27	0.001
T2D duration (month)	24 ± 9.1	NA	NA
MoCA score	24.94 ± 2.74	28.95 ± 1.05	<0.001
MMSE	28.24 ± 1.14	29 ± 0.62	n.s.

MoCA but not MMSE identify cognitive impairment in subjects with morbid obesity.

**Table 2:** The scores of the different domains evaluated by the MoCA test.

MoCA Domains	Obese Patients	Controls	*p*
Visuo Spatial Executive Function	4.0 ± 0.791	4.93 ± 0.26	0.001
Naming	2.94 ± 0.243	3.00 ± 0.00	n.s.
Attention	4.41 ± 1.73	5.67 ± 0.62	0.01
Language	2.71 ± 0.588	2.91 ± 0.32	n.s.
Abstraction	1.71 ± 0.772	1.96 ± 1.92	n.s.
Delayed Recall	3.14 ± 1.581	4.11 ± 1.31	0.015
Orientation	6.00 ± 0.00	6.00 ± 0.00	n.s.
Total	24.94 ± 2.74	28.95 ± 1.05	<0.001

Gaze fixation assessment but not retinal sensitivity is impaired in subjects with morbid obesity and is related to MoCA (Montreal Cognition Assessment Test).

**Table 3:** Microperimetry parameters between obese patients and control.

Microperimetry Parameters	Obese Patients	Controls	*p*
*N*	50	30	NA
Sensitivity (dB)	27.6 ± 3.81	29.28 ± 1.39	n.s.
Fixation P1 (%)	78.48 ± 26.16	97.18 ± 3.2	0.001
Fixation P2 (%)	90.31 ± 17.84	98.40 ± 4.39	0.001
BCEA63	1.50 ± 1.32	0.40 ± 0.34	0.001
BCEA95	8.33 ± 4.66	3.01 ± 2.01	0.021
Reliability index	93.52 ± 11.75	95.45 ± 15.07	n.s.

**Table 4:** Comparison between MO patients with T2D and without T2D.

Characteristics	Obese Patients with T2D	Obese Patients Without T2D	*p*
*N*	24	26	NA
Gender (female %)	70.37	71.42	n.s.
Age (years)	47.63 ± 8.62	44.33 ± 11.41	n.s.
BMI (kg/m^2^)	44.36 ± 5.4	44.70 ± 5.66	n.s.
HOMA-IR	8.34 ± 2.08	4.71 ± 2.047	0.001
HbA1c (%Hb DCCT)	6.62 ± 1.3	5.33 ± 0.28	0.001
T2D duration (month)	24 ± 9.1	NA	NA
MoCA score	25.5 ± 2.61	24.44 ± 2.92	n.s.
MMSE	28.1 ± 1.53	28.09 ± 1.78	n.s.
Retinal sensitivity (dB)	27.76 ± 2.45	27.59 ± 4.58	n.s.
Fixation P1 (%)	82.48 ± 23.59	78.19 ± 27.6	n.s
Fixation P2 (%)	92.00 ± 17.21	89.54 ± 18.04	n.s.
BCEA63	1.47 ± 1.44	1.51 ± 1.25	n.s.
BCEA95	8.14 ± 4.5	8.46 ± 4.85	n.s.
Reliability index	91.52 ± 14.75	92.65 ± 12.96	n.s.
Education level	7.08 ± 0.33	7.16 ± 0.28	n.s.

**Table 5:** HOMA-IR is a predictor of cognitive impairment in a multiple regression analysis model.

Coefficients(a)										
**Model**		**Unstandardized Coefficients**		**Standardized Coefficients**	**t**	**Sig.**	**95% Confidence Interval for B**		**Collinearity Statistics**	
		**B**	**Std. Error**	**Beta**			**Lower Bound**	**Upper BOUND**	**Tolerance**	**VIF**
1	(Constant)	25.200	3.734		6.749	0.000	17.684	32.715		
	Gender	−0.503	0.776	−0.084	−0.648	0.520	−2.065	1.060	0.869	1.151
	Age	−0.063	0.034	−0.242	−1.823	0.075	−0.132	0.007	0.831	1.203
	Type 2 Diabetes	−0.169	0.286	−0.076	0.589	0.559	−0.745	0.408	0.888	1.127
	HbA1c levels	0.233	0.375	0.081	0.620	0.538	−0.523	0.989	0.869	1.151
	BMI	0.078	0.050	0.209	1.574	0.122	−0.022	0.179	0.836	1.196
	HOMA-IR	−0.149	0.053	−0.375	−2.836	0.007	−0.255	−0.043	0.842	1.188

a. Dependent Variable: MoCA score.
